# Integration of heterogeneous functional genomics data in gerontology research to find genes and pathway underlying aging across species

**DOI:** 10.1371/journal.pone.0214523

**Published:** 2019-04-12

**Authors:** Jason A. Bubier, George L. Sutphin, Timothy J. Reynolds, Ron Korstanje, Axis Fuksman-Kumpa, Erich J. Baker, Michael A. Langston, Elissa J. Chesler

**Affiliations:** 1 The Jackson Laboratory, Bar Harbor ME, United States of America; 2 The University of Arizona, Molecular and Cellular Biology, United States of America; 3 Baylor University, Waco, TX, United States of America; 4 The Jackson Laboratory Nathan Shock Center of Excellence in the Basic Biology of Aging, The Jackson Laboratory, Bar Harbor, ME, United States of America; 5 University of Tennessee Knoxville, Knoxville, TN, United States of America; Foundation for Research and Technology-Hellas, GREECE

## Abstract

Understanding the biological mechanisms behind aging, lifespan and healthspan is becoming increasingly important as the proportion of the world's population over the age of 65 grows, along with the cost and complexity of their care. BigData oriented approaches and analysis methods enable current and future bio-gerontologists to synthesize, distill and interpret vast, heterogeneous data from functional genomics studies of aging. GeneWeaver is an analysis system for integration of data that allows investigators to store, search, and analyze immense amounts of data including user-submitted experimental data, data from primary publications, and data in other databases. Aging related genome-wide gene sets from primary publications were curated into this system in concert with data from other model-organism and aging-specific databases, and applied to several questions in genrontology using. For example, we identified *Cd63* as a frequently represented gene among aging-related genome-wide results. To evaluate the role of *Cd63* in aging, we performed RNAi knockdown of the *C*. *elegans* ortholog, *tsp-7*, demonstrating that this manipulation is capable of extending lifespan. The tools in GeneWeaver enable aging researchers to make new discoveries into the associations between the genes, normal biological processes, and diseases that affect aging, healthspan, and lifespan.

## Introduction

The population of individuals aged 65 and over is projected to be approximately 83.7 million in 2050, almost double its estimated number of 43.1 million in 2012 [1]. Aging affects the entire organism, with age-associated decline occurring across organ systems and within distinct tissues and cell-types. One approach to identifying shared mechanisms of aging is to make systems-level comparisons at the molecular level. To discover mechanistic pathways that may have applications in prediction and extension of life- and health-span, researchers have been analyzing the biological process of aging using a variety of high-throughput technologies including genome sequencing, RNAseq, proteomics, and structural biology. These studies make use of diverse animal model systems, such as nematodes, fruit flies, mice and rats to characterize natural aging, and drugs or environmental interventions that affect longevity, but all too often these large data sets remain underutilized and the potential to find convergent evidence for the role of molecular mechanisms in age-related phenomena are lost. By integrating data from multiple studies, one can find such evidence, identifying new potential mechanisms of healthy aging for hypothesis testing and validation.

Several representative applications merit an integrative genomics approach to aging. One application is to determine which molecular and cellular factors responsible for the process of cellular senescence also underlie functional cognitive decline. Cellular senescence is an anti-cancer and wound healing mechanism characterized by arrested cellular proliferation and secretion of pro-inflammatory cytokines, chemokines, growth factors, and proteases (the senescence associated secretory phenotype, or SASP). Senescent cells accumulate with age in many tissues, where the SASP promotes chronic inflammation and exacerbates age-associated degeneration and hyperplasia. Recent evidence suggests that neurological aging and neurodegeneration are accompanied by an accumulation of secretory cells in brain, suggesting that cellular senescence may contribute to brain aging [2] through a shared mechanism. Overlapping mechanisms can be detected using functional genomics studies of both the biology of cellular senescence and cognitive aging.

A second application is to determine what gene products are common to, two different disease states, as has been observed for obesity and dementia [3]. An integrative functional genomics approach can be used to determine the common molecular and cellular bases of these seemingly different disease conditions. A third application is to identify molecular mechanisms common to multiple anti-aging interventions. Dietary restriction has been shown to increase lifespan in a variety of species [4]. An ongoing effort is underway to identify drugs that mimic the beneficial outcomes by targeting molecular pathways downstream of dietary restriction. A number of pharmacological compounds have been identified that are capable of extending the life span of invertebrates and rodents, and represent potential dietary restriction mimetics [5]. Using heterogeneous data integration, large data sets can be investigated to determine whether a common network of genes underlies both approaches to life extension, that of dietary restriction or pharmacological intervention. Finally, in a fourth application one may determine whether a gene(s) function in aging has an evolutionarily conserved role. Relying upon the assumptions of phylogenomics, whereby an ortholog of one species has the same function in ancestral species, we can use large scale data from multiple species to understand conserved mechanisms of disease. Many additional application of global gene set integration exist.

With the goal of addressing these types of questions, and in supporting the research community in applying integrative functional genomics analysis of datasets obtained across different types of experiments and organisms, we created GeneWeaver [6]. This freely available web-based software system for the collection and analysis of functional genomic experiments allows for the rapid and easy integration of large quantities of heterogeneous data. Here we present the result of curating genomic studies from the aging literature which allowed us to then apply GeneWeaver’s suite of simple integrative functional genomics tools to identify relations among biological pathways of aging (Table 1).

**Table 1:** Summary statistics on a search of GeneSets, genes, or ontologies for “senescence OR aging OR longevity”.

Types of GeneWeaver Data	Number of GeneSets
Aging-related sets	1,655
GO^1^ term-based	71
MP^2^ term-based	63
HP^3^ term-based	109
MeSH^4^ term-based	231
QTL^5^		409
GWAS^6^		5
Gene expression	169
Drug-regulated gene sets [50]	253
Genes co-expressed to the aging phenotype	18

^1^GO (Gene Ontology)

^2^MP (Mammalian Phenotype Ontology)

^3^HPO (Human Phenotype Ontology)

^4^MeSH (Medical Subject Headings)

^5^QTL (Quantitative Trait Loci)

^6^GWAS (Genome Wide Association Study).

The data from these large-scale approaches provide a means to compare and contrast aging across model systems, tissues and interventions. Unfortunately, much data is presented in a non-computable format such as in primary publications, or in diverse and Balkanized data resources that require extensive efforts for integrative reanalysis. For example, the supplemental tables in publications often include lists of genes or gene networks that are analyzed in the publication but never fully integrated with existing data collected in related contexts. There have been several successful efforts to address these problems in the aging field, with large-scale, aging-related -omics databases including GenAge [7] and AgeFactDB [8]. These are excellent data repositories, however, it remains difficult to integrate heterogeneous data across studies, organisms and experiment types. These and many other resources bring the data together in one location but lack the tools and algorithms necessary to operate on the data as a whole. Across research disciplines, but certainly also within the aging field, readily accessible data is dwarfed by the immense volume of published but uncurated data. Further, the volume of uncurated data is increasing even more rapidly. In order to harness the knowledge that lies dormant in large published datasets, two steps are necessary: 1) published data must integrated; and 2) publicly accessible tools must be developed that are capable of integrating data from multiple sources into large-scale analysis.

Here we have curated studies from the aging literature and utilized integrative functional genomics in GeneWeaver to address four questions related to aging by analyzing these large-scale, complex sets of data: 1) to identify molecular relations between cellular senescence and functional cognitive decline, 2) to examine the intersection between comorbid disease states, 3) to identify new druggable targets for longevity, and 4) to examine cross-species translation of age-related processes.

## Methods and materials

### Integrative genomics in GeneWeaver.org

GeneWeaver is both a database of functional genomics gene sets and a suite of combinatorial and statistical tools that enable users to operate on these sets. GeneWeaver was designed to integrate large-scale genomic studies and houses these analytic tools and curated data from multiple species, ontological resources, and individual users in one central location. Users can upload sets of genes from personal or published experimental results that can then be made publicly available as part of the shared archive of gene sets in GeneWeaver, or analyzed privately. In the data archive, user submitted gene sets are integrated with gene sets from multiple sources including other individual users, publications with curated data, and large community data sources. Data resources incorporated include the drug-related gene database of the Neuroscience Information Framework (NIF) [9], GeneNetwork [10], Comparative Toxicogenomics Database (CTD) [11], Kyoto Encyclopedia of Genes and Genomes (KEGG) MeSH (Medical Subject Headings), Molecular Signatures Database (MSigDB), Online Mendelian Inheritance in Man (OMIM), and Pathway Commons. Data from these diverse resources are distilled into sets of genes within the GeneWeaver database. Additional gene sets in GeneWeaver are derived from data previously annotated to biological processes, disease states, or ontologies from model organism databases.

### Curation of the aging genomics literature in GeneWeaver

A search of PubMed for aging, cognitive decline and other relevant terms crossed with “genetic”, “gene expression”, “microarray”, “RNAseq” was used to identify publications which may contain gene sets relevant to age-related phenomena. Each publication was screened and relevant gene sets were entered into the database.

### Application of GeneWeaver analysis tools

GeneWeaver’s analysis tools were applied to the gene set database, including the curated literature. The tools support set operations and statistical analyses in a variety of user directed workflows. The specific workflows are described in the context of the results.

### Combine tool

The GeneWeaver “Combine” tool was used to create a set of genes representing the union of ontology annotation derived gene sets related to properties of cellular senescence and another set of genes containing genes experimentally related to functional decline such as genome-wide differential expression data related to aging-related cognition and memory phenotypes. The combine tool was used to obtain the union of all the genes within a selected gene set and provides a count as to the number of sets each gene is found in.

### Jaccard Similarity tool

GeneWeaver’s “Jaccard Similarity” tool was used on two genes sets to identify genes at the intersection of both senescence and functional decline. The “Jaccard Similarity” tool provides a pairwise comparison of the gene sets being analyzed. The resulting graph shows the overlap of genes in the sets using Venn diagrams. A Jaccard similarity coefficient is calculated by taking the size of the intersection of the two gene sets, and dividing by the number of unique genes available in the two sets. A value of 1.0 means perfectly overlapping, and 0 means no intersection. The “Jaccard Similarity” tool was again used to determine the similarity among mouse genes annotated to obesity and mouse genes annotated to abnormal learning and memory. A resampling strategy provides empirical p-values for results obtained using the Jaccard similarity tool.

### Assessing statistical significance of gene set overlap

Permutation testing (n = 2000, unless otherwise stated) was used to determine the probability of discovering at least one gene among a number of gene set intersections. A similar methodology, rigorously described by Real and Vargas [12] is used by the Jaccard similarity tool to assess the significance of Jaccard coefficients. Briefly, a null distribution of intersection cardinalities is generated from randomly sampling current gene lists from GeneWeaver. The sampling procedure accounts for cross-species relationships among sets i.e., homologous associations. Significance is then assessed using the cumulative probability of encountering at least one gene among N gene sets of given sizes and species.

### View similar gene sets

In order to identify other compounds that affect the same set of genes underlying the effects of caloric restriction, the “View Similar GeneSets” tool was used. Viewing similar gene sets within GeneWeaver compares the composition of the gene set of interest with all gene sets in the database and returns a ranked list based upon the magnitude of the Jaccard similarity of the top 250 gene sets most similar to the gene set of interest.

### GeneSet Graph

To identify the most highly connected gene within a group of gene sets related to aging, the “GeneSet Graph” tool was used. This tool presents a bipartite graph visualization of genes and gene sets. Genes are represented by elliptical nodes, and gene sets are represented by boxes. The least-connected genes are displayed on the left, followed by the gene sets, then the more-connected genes in increasing order to the right. Genes and gene sets are connected by colored lines to show what genes are in which gene sets. A degree threshold is applied on the gene partite set to reduce the graph size.

### Boolean Algebra tool

The “Boolean Algebra” tool was used to identify the genes at the intersection of two gene sets, one from each species tested for caloric restriction. A new gene set was created from the genes within this intersection. This approach allow the rapid determination of new relationships between gene sets and the creation of new gene sets based upon these findings. This same approach was repeated to compare gene sets related to differential drug treatment and overlap between both interventions.

### STRING

The set of genes determined to be at the intersection of functional decline and senescence were uploaded into the STRING 11.0 database (https://string-db.org/) of predicted and known protein-protein interactions. A graphical representation of the functional associations known between the encoded proteins was produced. STRING produces a PPI enrichment p-value which tests the likelihood that a set of proteins have more interactions among themselves than what would be expected from a random set of proteins of similar size, drawn from the genome. Such a significant enrichment indicates that the proteins are at least partially biologically connected as a group.

### Over-representation test

The PANTHER 14.0 was used to assess the over-representation of different Gene Ontology Molecular Processes within sets of genes created in GeneWeaver[13]. The data was analyzed with Fisher’s Exact test and corrected for multiple testing.

### Ingenuity pathway analysis

The genes at the intersection of obesity and dementia were uploaded to the Ingenuity Pathway Analysis software v01-07. This application maps genes to existing molecular pathways based on built in algorithms and gives predictions of for the likelihood of the observed enrichment.

### Worm lifespan

*C*. *elegans* experiments were carried out using wild-type (N2) worms, originally obtained from Matt Kaeberlein at the University of Washington. Worms are stored long-term at -80°C, freshly thawed every 3 months, and never allowed to starve. The RNAi feeding clone targeting *tsp-7* was obtained from the Ahringer library [14] and the target sequence confirmed prior to use. *C*. *elegans* lifespan were conducted at 25°C according to standard protocols [15]. Briefly, experiments were conducted on nematode growth media (NGM) plates containing 1 mM Isopropyl β-D-1-thiogalactopyranoside (IPTG) to activate production of RNAi transcripts and 25 μg/mL carbenicillin to select RNAi plasmids and seeded with live *E*. *coli* (HT115) containing either *tsp-7* or *empty vector (EV)* RNAi feeding plasmids. Worms were age-synchronized via timed egg laying and transferred to plates containing 50 μM 5-fluorodeoxyuridine (FUdR) at the L4 larval stage to prevent reproduction. Worms were fed *E*. *coli* expressing the indicated RNAi sequence starting at egg and continuing throughout lifespan. Worms were scored as alive or dead every 1–2 days until all animals had died and transferred to new plates in the case of fungal contamination. Experiments were conducted in triplicate, with each replicate containing ~105 (~35 worms/plate on 3 plates), with each replicate showing a similar survival pattern. We used a log-rank test to compare survival differences between *EV(RNAi)* and *tsp-7(RNAi)* for each experiment individually and for survival data pooled across experiments. Kaplan-Meier survival curves are presented for pooled data. We conducted all survival analyses using the “survival” package in R.

### *Cd63* in human GWAS

GWA summary statistics were retrieved from LD Hub [16] and made available through an aging study [17] which made use of data from the UK Biobank [18]. We examined three longevity phenotypes: mother’s age at death, father’s age at death, and the combined phenotype of parent’s age at death. Genomic positions for each SNP were converted to the hg38/GRCh38 genome build using the UCSC liftOver tool. SNPs and positions that could not be converted were discarded. SNP reference identifiers were updated to the latest (at the time of writing) NCBI dbSNP build—version 150. SNPs without a canonical reference identifier (rsID) were discarded. Summary statistics were filtered to only include significant (p < 0.05) SNPs. Variant annotations from Ensembl v. 91 were used to annotate SNPs to the genomic features they occur in. The UK Biobank aging dataset contained five SNPs which occur in CD63. We also examined SNPs immediately downstream and upstream of CD63. There were nine downstream and 11 upstream variants. The false discovery rate, q-value was calculated using the R qvalue package [19].

## Results

### Identifying molecular relations between the biological processes of cellular and replicative senescence and cognitive functional decline *in vivo*

**Fig 1:**
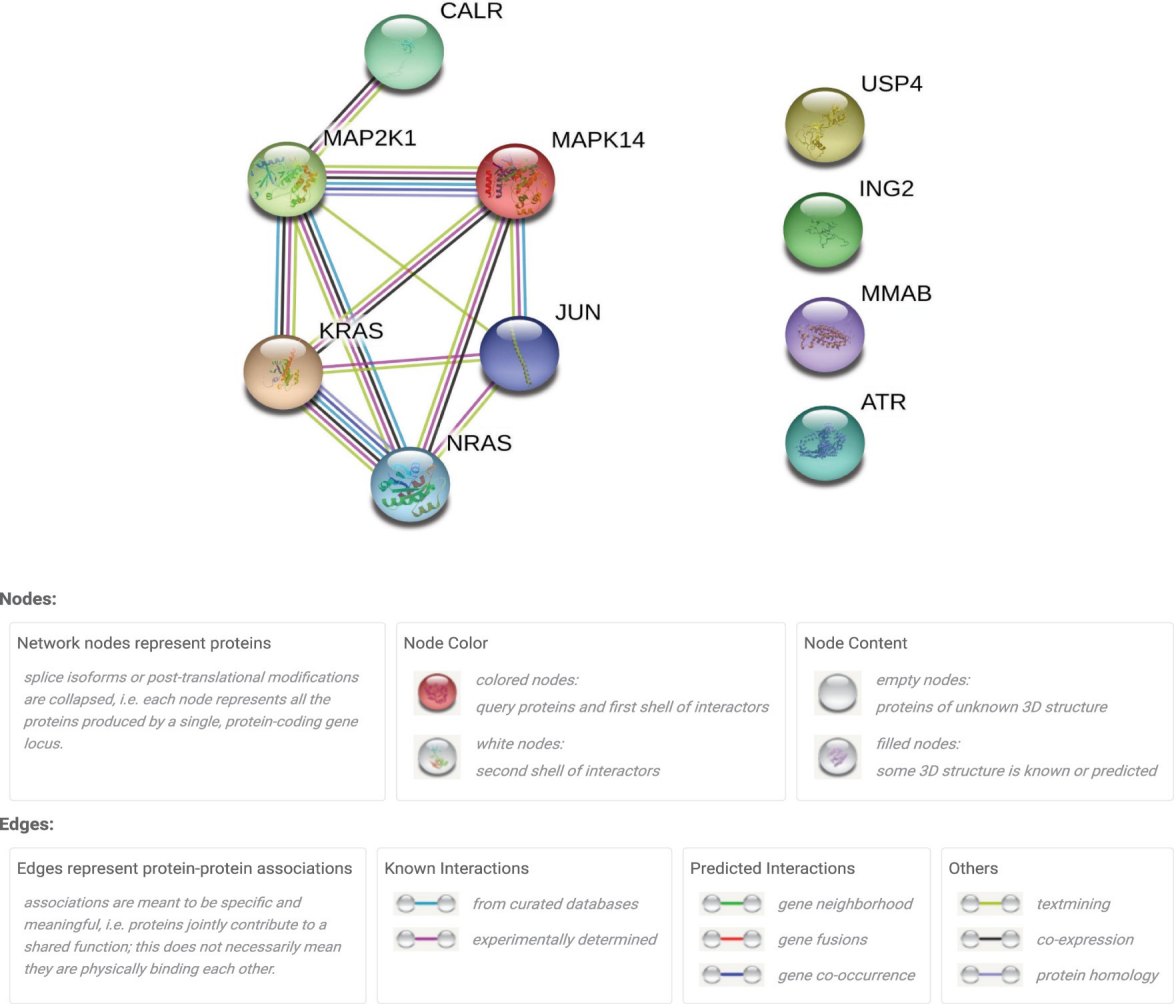
Conserved pathways between process of cellular senescence and functional decline. A group of 10 genes was identified as common to functional decline and senescence. Many of these genes interact in the MAP kinase pathway, as shown by this protein-protein interaction plot from STRING.

GeneWeaver was queried to identify gene sets related to the biological process of cellular senescence and phenotype of cognitive decline. A conservative combined set of 92 genes (GS222630) unambiguously related to cellular and replicative senescence was created from the union of model organism ontology annotations to the terms MP:0008007 abnormal cellular replicative senescence (GS164391), and the gene ontology biological processes of cellular senescence (GO:0090398, GS308156) and replicative senescence (GO:0090399, GS307811). A second set of 1,286 “experimentally observed” genes (GS222631), resulted from the complete intersection of genes from eight published studies on differentially expressed genes associated with either *in vivo* studies of functional decline or aging-related cognition and memory phenotypes S1 Table [20–26]. The gene sets from each publication contain the genes the authors determined to be significantly different in each study. Using GeneWeaver’s “Jaccard Similarity” tool on these two resulting sets of genes, an intersection set of ten genes common to both the biological process of cellular and replicative senescence and the phenotype of functional decline was obtained. This pair of gene sets had a Jaccard coefficient 0.007 and a permutation based suggestive p-value < 0.09 based upon 1,500 permutation tests. This indicates that only a subset of cognitive decline related genes overlap with cellular senescence and only a subset of cellular senescence genes overlap with cognitive decline. To further interpret the function of this overlapping subset, we performed downstream pathway analyses. Ingenuity Pathway Analysis (IPA) revealed that the intersection is significantly enriched with UVC-MAP kinase pathway members (likelihood 5.52 x 10^12^, Fisher’s Exact Test, S2 Table). The ten genes were analyzed for known and predicated protein-protein interactions using the STRING database of protein-protein relations (https://string-db.org/) and shown to have significantly more interaction than would be expected (p<0.00284) from a randomly chosen set of proteins of similar size, drawn from the genome. Within this module are two physically interacting pairs supported by experimental evidence, MAP2KL-MAPK14 and KRAS-HRAS (Fig 1). Identification of these proteins and this cellular pathway within eight different functional genomic experimental data sets related to cognitive decline and genes related to the biological processes of cellular and replicative senescence suggest that functional decline and cellular and replicative senescence share key central, conserved cellular signaling pathways despite a lack of global similarity of cognitive decline and cellular senescence. We report this finding to demonstrate this general and extendable approach for selecting a curated ontological association for one biological process and identifying a conserved molecular function pathway from within noisy heterogeneous functional genomics data of differing related biological phenomena.

### Identifying a common molecular basis of obesity and dementia

**Fig 2:**
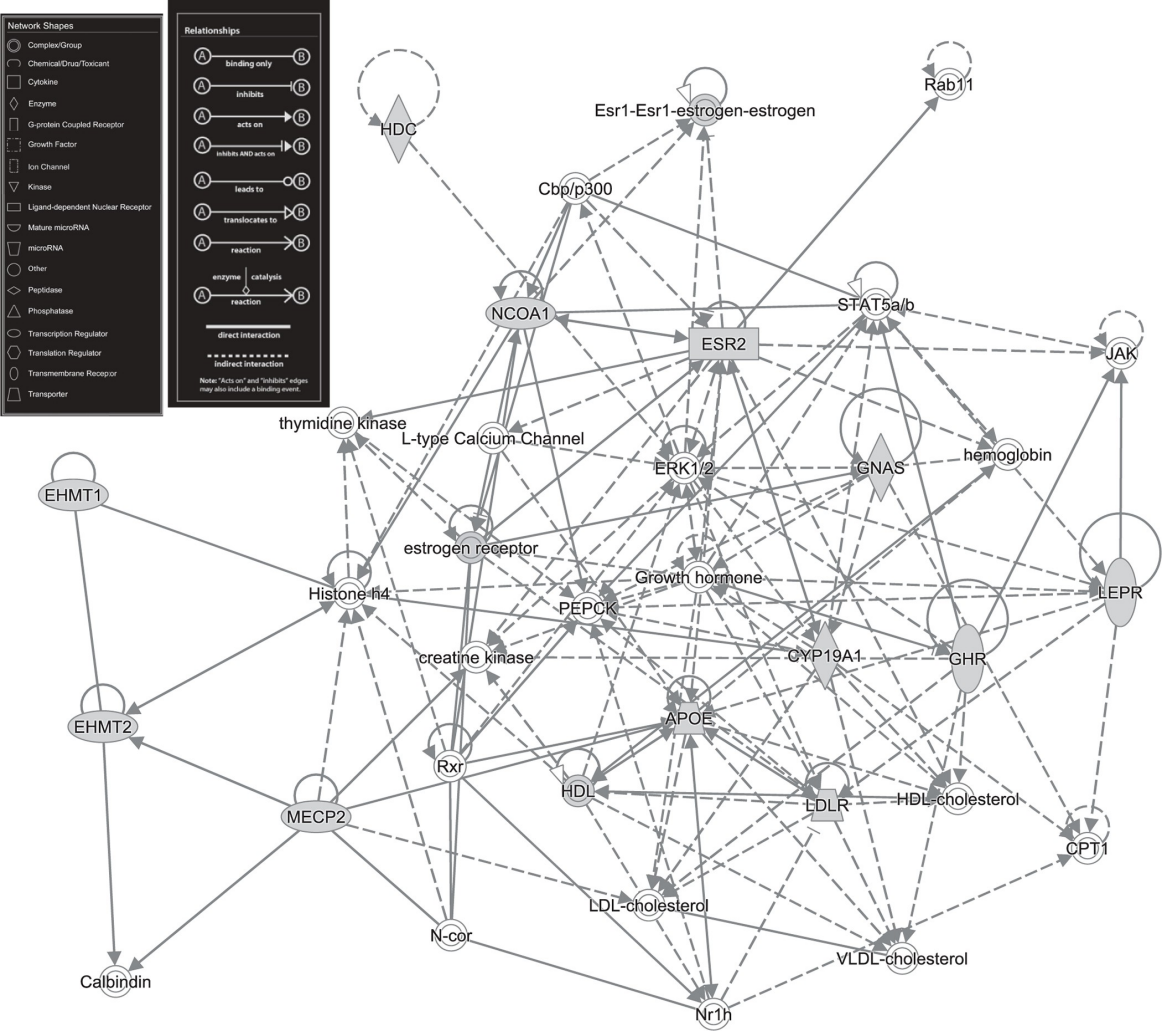
Pathways common to obesity and dementia. Ingenuity pathway analysis demonstrates that 12 of the 15 genes identified to be at the intersection of two often co-occurring conditions, obesity and dementia, map to one pathway.

To identify possible common molecular mechanisms underlying obesity and dementia, GeneWeaver’s database was searched to identify relevant gene sets, in this case, phenotypic alleles annotated in model organism databases to the Mouse Phenotype Ontology. Using the “Jaccard Similarity” tool in GeneWeaver, the similarity among mouse genes annotated to GS165384 MP:0001261 obesity (98 genes) and mouse genes annotated to GS169386 MP:0002063 abnormal learning and memory (585 genes) was determined; this overlap contained 15 genes (with a Jaccard coefficient of 0.0225 and p <0.002). Ingenuity Pathway Analysis (IPA) showed the most highly significant pathways included “Behavior, Connective Tissue Development and Function, Tissue Morphology” which contained 12 of the 15 genes (likelihood 1 x 10^31^, Fisher’s exact test; Fig 2; and S3 Table). Emerging research supports the role of adipokines in adipose tissue dysfunction where they exert effects upon neurodevelopment and cognition across life span [27]. Identification of this pathway identifies potential translational targets for therapies for treating or preventing dementia in the aging obese population.

### Finding pathways that underlie both life-extension drugs and dietary restriction that are conserved between *Mus musculus* and *Drosophila melanogaster*

To determine whether a common network of genes underlies two lifespan extending interventions across species, that of dietary restriction or pharmacological intervention, we compared associated genomic data using GeneWeaver. Caloric restriction has been repeatedly shown in diverse organisms to extend the length of life. We chose to compare two model organisms evolutionarily separated by 500 million years, mouse and fly. Using data from a functional genomic studies of mouse (GS222634, [28]) or fly (GS213271, [29]) which examined gene expression in response to caloric restriction, the intersection of the two sets—one from each species—was taken using the “Boolean Algebra” tool in GeneWeaver. This intersection produced 35 cross-species homologs. We suggest that these 35 genes co-occurring in both genome wide studies of two different species in response to caloric restriction (J = 0.0118, p<0.002) represent the evolutionarily conserved targets of caloric restriction related to aging. Several drugs have been shown to extend lifespan in both species (see DrugAge database [30]) as representative drugs we selected Sirolimus-rapamycin, in mouse [31, 32], and 3,5,4'-trihydroxystilbene resveratrol, in fly [33] and using data from the CTD, a database of manually curated information about chemical gene/protein interactions, we sought to create a set of representative life extending drug target genes, one set of genes was created for each drug (GS122305 and GS126476). Using data from each drug the intersection of the two sets was taken using the “Boolean Algebra” tool in GeneWeaver. The result contained 181 genes common to both drugs (J = 0.0807, p<0.002). To determine whether the 35 caloric restriction genes and these two drugs share a common molecular mechanism of action, the set of 35 homologs was overlapped with the data set produced from the two-way intersection of “life-extending drugs.” HSPA1A, an HSP70 complex member, was the one gene product (p = 0.0419) common to all pathways. This is consistent with previous aging studies suggesting that the abundance of HSP70 complex members decreases with aging [34, 35], and identifying SNPs that are linked to HSP70 genes and are associated with longevity [36, 37].

This approach identified a gene product, HSPA1A, common to dietary restriction and two known drugs that influence life span. To identify additional drugs/chemicals that could also function to target gene products that underlie the beneficial effects of dietary restriction we used the GeneWeaver “View Similar GeneSets” tool to identify gene sets that are associated with other drugs that overlap with our set of 35 aging gene homologs. The top ten compounds interacting with any of the 35 genes of interest are shown in Table 2. These compounds can now be investigated for their direction of effect, as they may either shorten or extend lifespan, depending upon the direction of gene expression change they induce, and depending on the results of the corresponding diet-restricted study.

**Table 2:** The top ten compounds known to interact with any of the 45 genes found at the intersection of cross-species dietary restriction aging studies.

Compound	ID	Target
α-[(S)-(Phosphonomethyl)amino]-3-dib enzofuranpropanoic acid	MeSH:C404932	ECE1
Cyclofenil	MeSH:D003506	SLC2A1
desmethylmisonidazole	MeSH:C009997	POR
flavins	MeSH:D005415	POR
fluorodeoxyglucose F18	MeSH:D019788	SLC2A1
misonidazole	MeSH:D008920	POR
3-(3-cyclohexyl-1-(2-(dimethylamino)-2-oxoethyl)-6-(4-methyl-5-oxo-4,5-dihydro-1,2,4-oxadiazol-3-yl)-1H-indol-2-yl)-N,N-dimethylbenzamide	MeSH:C544762	ELOVL6
apaziquone	MeSH:C060817	SLC2A1
laurates	MeSH:D007848	POR
ametryne	MeSH:C100057	POR

### Cross-species translation of age-related processes

**Fig 3:**
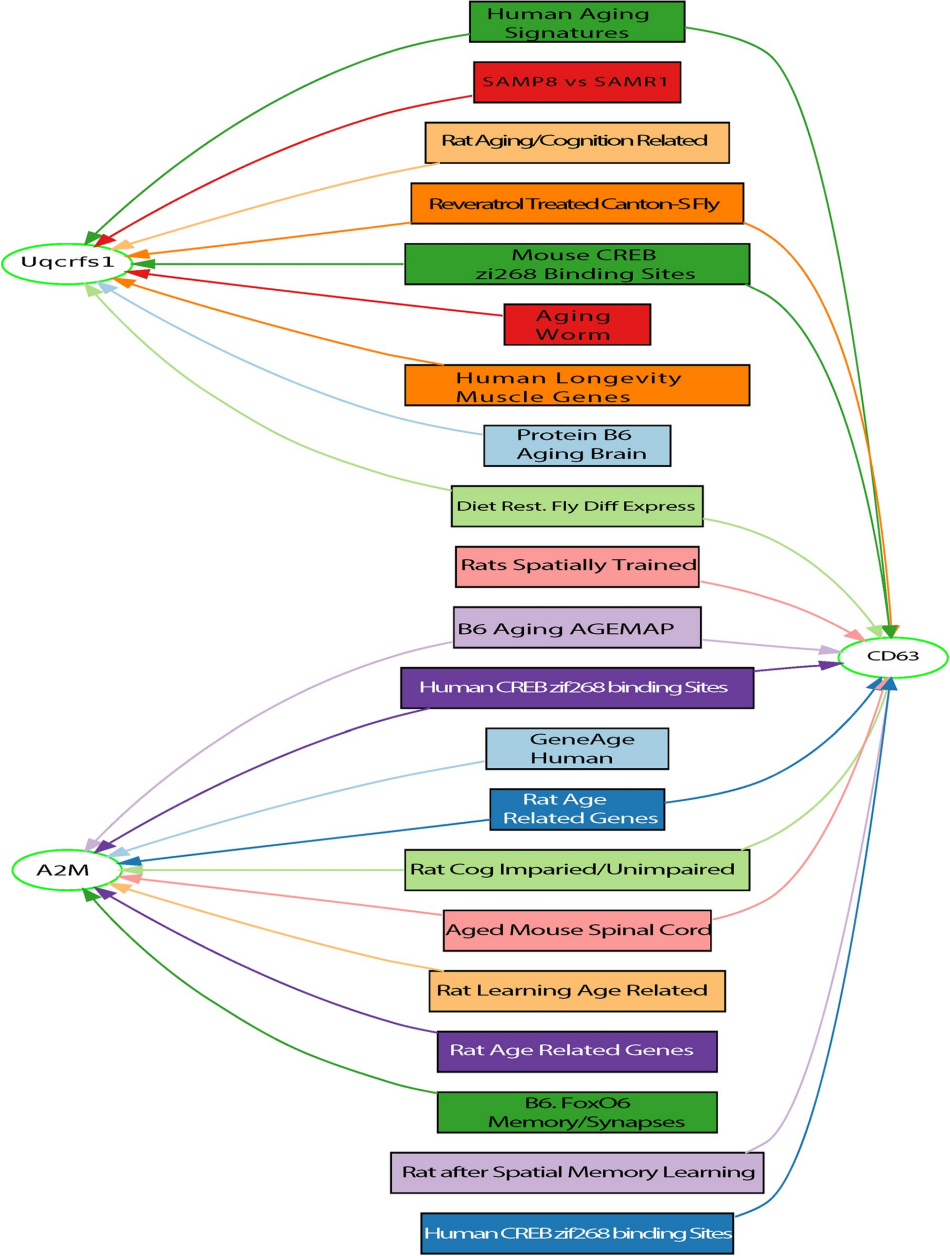
GeneSet graph of the most highly connected genes from 73 gene sets from six different species. The GeneSet Graph Tool presents a partitioned display of genes and GeneSets. Genes are represented by elliptical nodes, and GeneSets are represented by boxes. The least-connected genes are displayed on the left, followed by the GeneSets, then the more-connected genes in increasing order to the right. Genes and GeneSets are connected by colored lines to show what genes are in which GeneSets.

**Fig 4:**
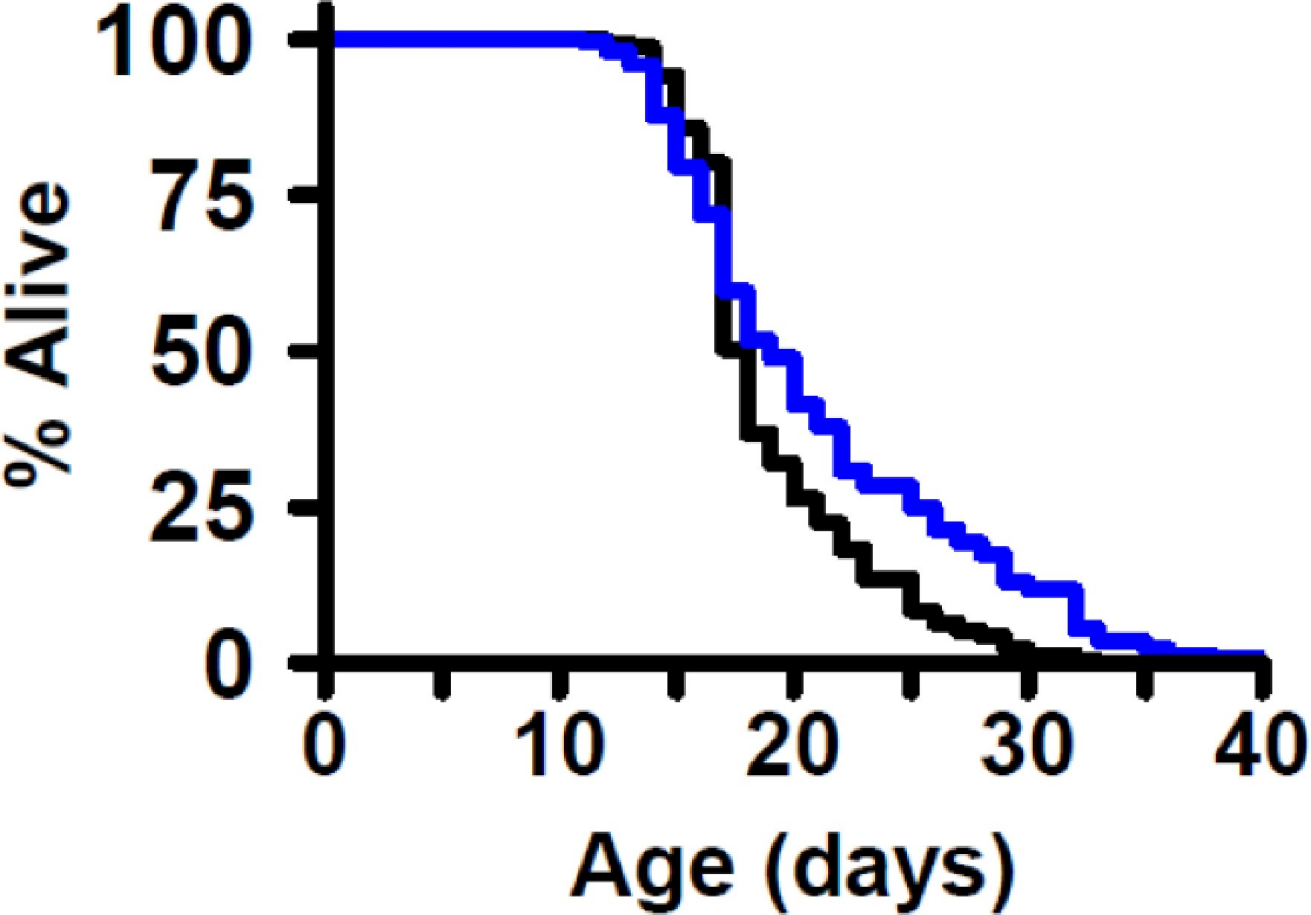
RNAi knockdown of *tsp-7* increases worm lifespan. Survival curves *C*. *elegans* fed either *empty vector (EV) RNAi* (black, n = 317) or *tsp-7(RNAi)* (blue, n = 312).

To identify convergent evidence across species for genes involved in aging, we integrated data from a total of 73 aging-associated gene sets (S4 Table), derived from 31 publications across 6 species (yeast, worm, fly, rat, mouse, human), and from three web resources (GeneNetwork, GenAge [38], and GWAS Catalog (https://www.ebi.ac.uk/gwas/). Using the “GeneSet Graph tool” in GeneWeaver, we identified *Cd63* as the most highly connected gene (i.e. it was present in the largest number of sets of genes) (Fig 3). *Cd63* was present in 12 gene sets from seven publications across four species (fly, rat, mouse, and human; Table 3). The probability of finding at least one gene in a 12-way intersection, given the observed set sizes and species, is p < 0.0005 (permutations n = 2000). To validate *Cd63* as an aging gene, we knocked down the *C*. *elegans* ortholog, *tsp-7*, by feeding RNAi and observed a 10.5% extension of mean lifespan (19.0±4.0, n = 312 for *empty vector(RNAi)* vs. 21.0± 6.5 days, n = 317 for *tsp-7(RNAi)* at 25°C; p = 4.8e-7 by the log-rank test) (Fig 4, S5 Table). Manipulating *tsp-7* is thus sufficient to influence lifespan in at least one environmental context.

**Table 3:** The 12, of 73 gene sets that contain *Cd63*.

GeneSet Number	Species	GeneSet Name	Publication
213295	Human	Aging Signatures	[7]
213272	Fly	Resveratrol Canton-S	[29]
216489	Mouse	CREB zif268 binding sites	[51]
216491	Human	CREB zif268 binding sites		[51]
216492	Rat	CREB zif268 binding sites	[51]
218922	Rat	Aged SpatiallyTrained	[52]
215692	Mouse	Aged Spinal Cord	[53]
213041	Mouse	B6 Aging AGEMAP	[53]
213271	Fly	Diet-restricted Diff Expression		[29]
137847	Rat	Cognitive Impaired vs Unimpaired		[54]
218921	Rat	Age-related Genes	[55]
218978	Rat	Spatially Trained	[52]

Further inspection of the same GeneSet graph (Fig 3) showed that ubiquinol-cytochrome c reductase, Rieske iron-sulfur polypeptide 1 (*Uqcrfs1*) is present in nine sets. The probability of finding at least one gene in a 9-way intersection, given the observed set sizes and species, is p < 0.0005 (permutations n = 2000). Rieske iron-sulfur proteins have been linked to aging in yeast [39] and worms [40, 41]. *Uqcrfs1* is involved in mitochondrial function [42], and a decline in activity and quality of mitochondria is associated with age-related diseases [43]. The third highest degree gene on that same graph, with equivalent number of connections (9) and significant p-value (p<0.0005) is alpha-2-macroglobulin (A2M). A2M has a well-characterized role in Alzheimer’s disease and aging in multiple species [44–46]. Together, these results demonstrate the utility of integrative functional genomics to identify aging-related genes using integrative gene set analysis across multiple species.

To determine whether *Cd63* has been significantly associated with aging phenotypes in the recent large GWAS studies by the UK Biobank [17, 18] we downloaded data from LD Hub and looked for association of SNP in or *near Cd63* with age related phenotypes (age of father’s death, age of mothers death etc). The UK Biobank aging dataset contained five SNPs which occur in CD63. Three (rs2231462, rs142309837, rs3138132) of these SNPs are 5’ UTR variants, one (rs2231464) is an intron variant, and another (rs35746357) is a noncoding transcript exon variant. However, none of these were significant for any of the three aging phenotypes we examined. We also examined SNPs immediately downstream and upstream of CD63. There were nine downstream and 11 upstream variants, none of these were significant. The most significant upstream variant, rs144565701 which is roughly 4KB from *Cd63*, was for the “mother’s age at death" phenotype (rs144565701, p = 0.034, q = 0.85).

## Discussion

The growing number of studies and data in many fields, including ageing, requires the development of integrative and computational approaches to analyze the data for consensus and shared biological findings across conditions. Using GeneWeaver’s database and analysis tools to address questions in aging research we were able to identify genes common to cellular senescence and functional cognitive decline; to examine gene products at the intersection between obesity and dementia, to identify several potential druggable targets for investigation in longevity, and to identify and validate a cross-species age-related gene from convergent evidence. Our identification of the role for CD63 in aging would not have been made without this use of this large genomic analysis tool. CD63 in *C*.*elegans* is member of the tertaspanin family of proteins [47]. Tetraspanins are transmembrane scaffolding proteins involved in motility, cell adhesion, proliferation and activation. Recently we showed that knockdown of another tetraspanin in *C*.*elegans*, *tsp-3*, extends lifespan by >20% lifespan as well [48], suggesting that this protein family may be of broader interest in aging.

As more aging-related functional genomic data is generated and made public by scientists all over the world, integrative functional genomics strategies will allow efficient integration of each new study into the growing pool of meta-data and rapid analysis that leverages the diversity of data produced across species and technical disciplines. Here we used several analytical approaches available in GeneWeaver, while other applications of GeneWeaver are available beyond these examples, including prioritization of QTL positional candidates, integration of GWAS data with expression data, and identification of animal models of aging phenotypes based on their underlying biology. These and other applications can be readily executed in GeneWeaver by users and have been summarized in a recent publication [49]. Functional enrichment analyses at any level need to be performed and reported with caution and choosing to focus on a subset of the retrieved functions is always bound to introduce bias in downstream analyses. The GeneWeaver database continues to grow in both the number and the variety of gene-sets it contains. Investigators in the aging research community are encouraged to submit their own studies to this system. The utility, power and scope of integrative tools like GeneWeaver expand with each new user and dataset. GeneWeaver tools and data resources are in continued development that will allow for integration of heterogeneous pathway-centric data, integrating at the level of pathways and variants rather than at the level of genes, across experiments and species. Together, these advances in aging data resource aggregation and analytics will enable the aging research community to readily identify convergent molecular evidence for novel mechanisms of aging, healthspan and lifespan.

## Supporting information

S1 Table The sets of genes derived from eight published studies on differentially expressed genes associated with either functional decline or aging-related cognition and memory phenotypes. (XLSX)

S2 Table The pathways found to be significantly enriched in the IPA analysis of the 10 genes at the intersection of the ontological associations and the eight functional genomic studies. (XLSX)

S3 Table The pathways found to be significantly enriched in the IPA analysis of the 35 genes at the intersection of obesity and dementia. (XLSX)

S4 Table The 73 aging-associated gene sets used to identify Cd63 and associated publications. (XLSX)

S5 Table Raw individual experiment data and survival plots from the *C*. *elegans* fed either *empty vector (EV) or tsp-7(RNAi)*. (XLSX)
